# Linkage mapping in the oilseed crop *Jatropha curcas* L. reveals a locus controlling the biosynthesis of phorbol esters which cause seed toxicity

**DOI:** 10.1111/pbi.12092

**Published:** 2013-07-30

**Authors:** Andrew J King, Luis R Montes, Jasper G Clarke, Julie Affleck, Yi Li, Hanneke Witsenboer, Edwin van der Vossen, Piet van der Linde, Yogendra Tripathi, Evanilda Tavares, Parul Shukla, Thirunavukkarasu Rajasekaran, Eibertus N van Loo, Ian A Graham

**Affiliations:** 1 Centre for Novel Agricultural Products, Department of Biology, University of YorkYork, UK; 2 Biocombustibles de GuatemalaGuatemala Ciudad, Guatemala; 3 Keygene N.V.Wageningen, The Netherlands; 4 Quinvita GroupGhent, Belgium; 5 Plant Breeding Wageningen URWageningen, The Netherlands

**Keywords:** *Jatropha curcas*, linkage mapping, oilseed, phorbol esters, plant breeding

## Abstract

Current efforts to grow the tropical oilseed crop *Jatropha curcas* L. economically are hampered by the lack of cultivars and the presence of toxic phorbol esters (PE) within the seeds of most provenances. These PE restrict the conversion of seed cake into animal feed, although naturally occurring ‘nontoxic’ provenances exist which produce seed lacking PE. As an important step towards the development of genetically improved varieties of *J. curcas*, we constructed a linkage map from four F_2_ mapping populations. The consensus linkage map contains 502 codominant markers, distributed over 11 linkage groups, with a mean marker density of 1.8 cM per unique locus. Analysis of the inheritance of PE biosynthesis indicated that this is a maternally controlled dominant monogenic trait. This maternal control is due to biosynthesis of the PE occurring only within maternal tissues. The trait segregated 3 : 1 within seeds collected from F_2_ plants, and QTL analysis revealed that a locus on linkage group 8 was responsible for phorbol ester biosynthesis. By taking advantage of the draft genome assemblies of *J. curcas* and *Ricinus communis* (castor), a comparative mapping approach was used to develop additional markers to fine map this mutation within 2.3 cM. The linkage map provides a framework for the dissection of agronomic traits in *J. curcas*, and the development of improved varieties by marker-assisted breeding. The identification of the locus responsible for PE biosynthesis means that it is now possible to rapidly breed new nontoxic varieties.

## Introduction

*Jatropha curcas* is being developed as a perennial oilseed crop for cultivation in tropical and subtropical climates (King *et al*., 2009). As a member of the Euphorbiaceae (spurge family), it is also related to a number of other agronomically important crops including cassava (*Manihot esculenta*), rubber (*Hevea brasiliensis*), castor (*Ricinus communis*) and a number of minor oilseed crops including tung (*Aleurites fordii*) and Chinese tallow (*Triadica sebifera*). Although there is a history of small-scale cultivation of *J. curcas*, particularly on the Cape Verde islands (da Silveira, 1934), economical cultivation of *J. curcas* has currently not reached its full potential due to a number of factors, including lack of knowledge of best agronomic practices and the lack of available purpose-bred cultivars. This has resulted in unpredictable seed yields, ranging from 0.3 to 3.9 tonnes per hectare (Kalannavar, 2008; Mohapatra and Panda, 2011; Yang *et al*., 2010).

Another current disadvantage of using *J. curcas* as an oilseed crop is that most provenances produce toxic seed. The exception is the ‘nontoxic’ provenances which occur naturally. Within Mexico, seeds from these nontoxic provenances are consumed by the local population (after roasting) (King *et al*., 2009). Metabolite analysis has revealed that the only major difference between the toxic and nontoxic seeds of *J. curcas* is the presence of phorbol esters (PE) within the seeds (He *et al*., 2011; Makkar *et al*., 1998). These PE are not fully degraded by the heat processing steps normally used in the conversion of raw seed meal to animal feed (Aregheore *et al*., 2003; Makkar *et al*., 1998). The production of animal feed from *J. curcas* seed therefore requires an additional solvent extraction step to remove the PE (Brooker, 2009). Concerns have also been raised about handling *J. curcas* products containing PE, as these compounds have also been shown to be cocarcinogens (Hirota *et al*., 1988)—although not carcinogenic on their own, PE can promote tumour formation caused by exposure to some chemical carcinogens (Goel *et al*., 2007). The commercialization of seeds lacking PE is therefore desirable, as the seed meal could more readily be converted to animal feed using traditional processes, and any potential risks from handling PE-containing products are removed.

Compared with more established oilseed crops which have seen significant increases in seed yield through breeding and agronomy (Vollmann and Rajcan, 2010), research and development of *J. curcas* is still at a very early stage. However, our knowledge of the plant has improved vastly in recent years. The genetic diversity of *J. curcas* is now better understood; a number of studies have revealed that meso-America is the centre of genetic diversity for the species, and there is very little genetic variation in material cultivated outside of this region (Basha *et al*., 2009; He *et al*., 2011; Sun *et al*., 2008; Yi *et al*., 2010). These studies on genetic diversity have provided useful guidance into sources of material for breeding programmes. Perhaps the most significant advance is in the amount of DNA sequence information which has become available. As well as a published draft genome sequence (Hirakawa *et al*., 2012; Sato *et al*., 2011), a number of transcriptomic studies have also been conducted (Costa *et al*., 2010; King *et al*., 2011; Natajaran and Parani, 2011). Despite these advances in our understanding of the molecular biology and genetic diversity of *J. curcas*, molecular breeding for rapid improvement of desirable traits such as seed oil yield and nontoxic seed has been severely hampered by the lack of a genetic linkage map. At present, the only map available for *Jatropha* sp. is from an interspecific cross between *J. curcas* and *J. integerrima* (Wang *et al*., 2011). Here, we report a high-density genetic linkage map of *J. curcas*, created from four separate mapping populations, and containing over 500 codominant (SSR and SNP) markers distributed over 11 linkage groups. As a first step in demonstrating the utility of this map, we have identified a locus responsible for the synthesis of the toxic phorbol esters. This work lays the foundation for more rapid crop improvement by marker-assisted breeding and the creation of new nontoxic varieties of *J. curcas*.

## Results and discussion

### Development of codominant markers and the construction of an integrated linkage map from four F_2_ mapping populations

For the construction of linkage maps, codominant markers such as single nucleotide polymorphisms (SNP) and simple sequence repeats (SSR) are the most useful as they provide information on both alleles present in a diploid species such as *J. curcas*. At the commencement of this study, very few codominant markers were available for *J. curcas* and these were mainly in the form of SSR (Basha *et al*., 2009; Phumichai *et al*., 2011). To provide sufficient markers for the construction of a linkage map, additional SNP markers were obtained from genomic DNA using the CRoPS® technique (File S1). This is a complexity reduction technique that is used for obtaining reduced representation genomic DNA libraries and is an adaptation of the AFLP technique (van Orsouw *et al*., 2007). SNP markers were also mined from seed transcriptome sequence produced from both toxic (King *et al*., 2011) and nontoxic [A.J. King and I.A. Graham, unpublished data] developing seeds (File S2). SSR markers were developed using the FIASCO protocol (Zane *et al*., 2002), or by mining *J. curcas* genome sequence data (Hirakawa *et al*., 2012; Sato *et al*., 2011) using either WebSat (Martins *et al*., 2009) or Imperfect SSR Finder (Stieneke and Eujayl, 2007). The SSR sequences and the primers used are detailed in File S3. In order to allow the numbering of chromosomes to remain consistent with the previously published map of the *J. curcas* × *J. integerrima* interspecific cross (Wang *et al*., 2011), we included a number of markers which could be used as bridges between these maps (File S4). This was achieved by using markers which had previously been mapped on the interspecific cross, or physical mapping of markers from both maps onto scaffolds of the *J. curcas* genome sequence (Hirakawa *et al*., 2012; Sato *et al*., 2011).

**Figure 1:**
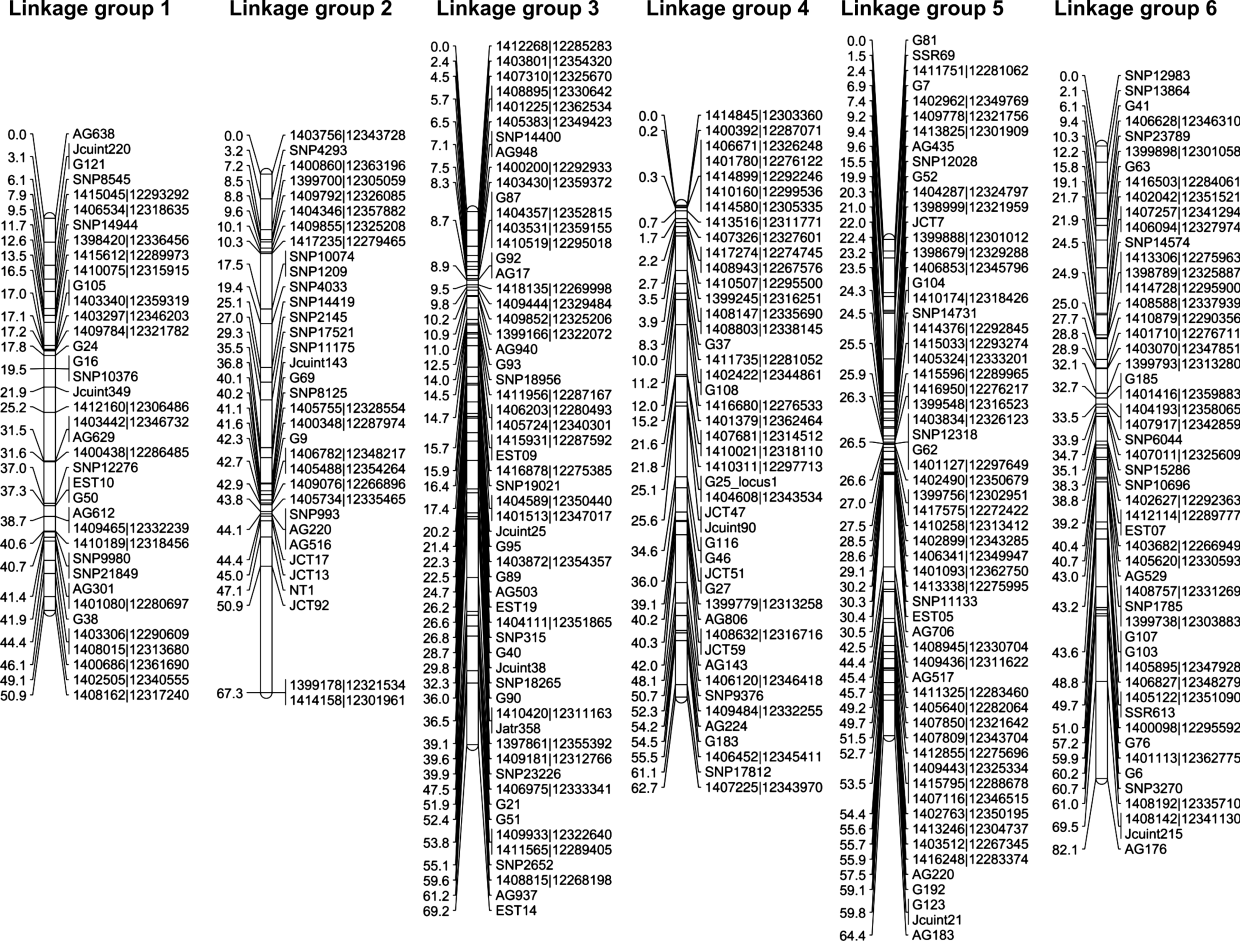
Integrated genetic map for linkage groups 1–6 of *Jatropha curcas* produced from four mapping populations. Linkage group positions are indicated in cM (Kosambi).

**Figure 2:**
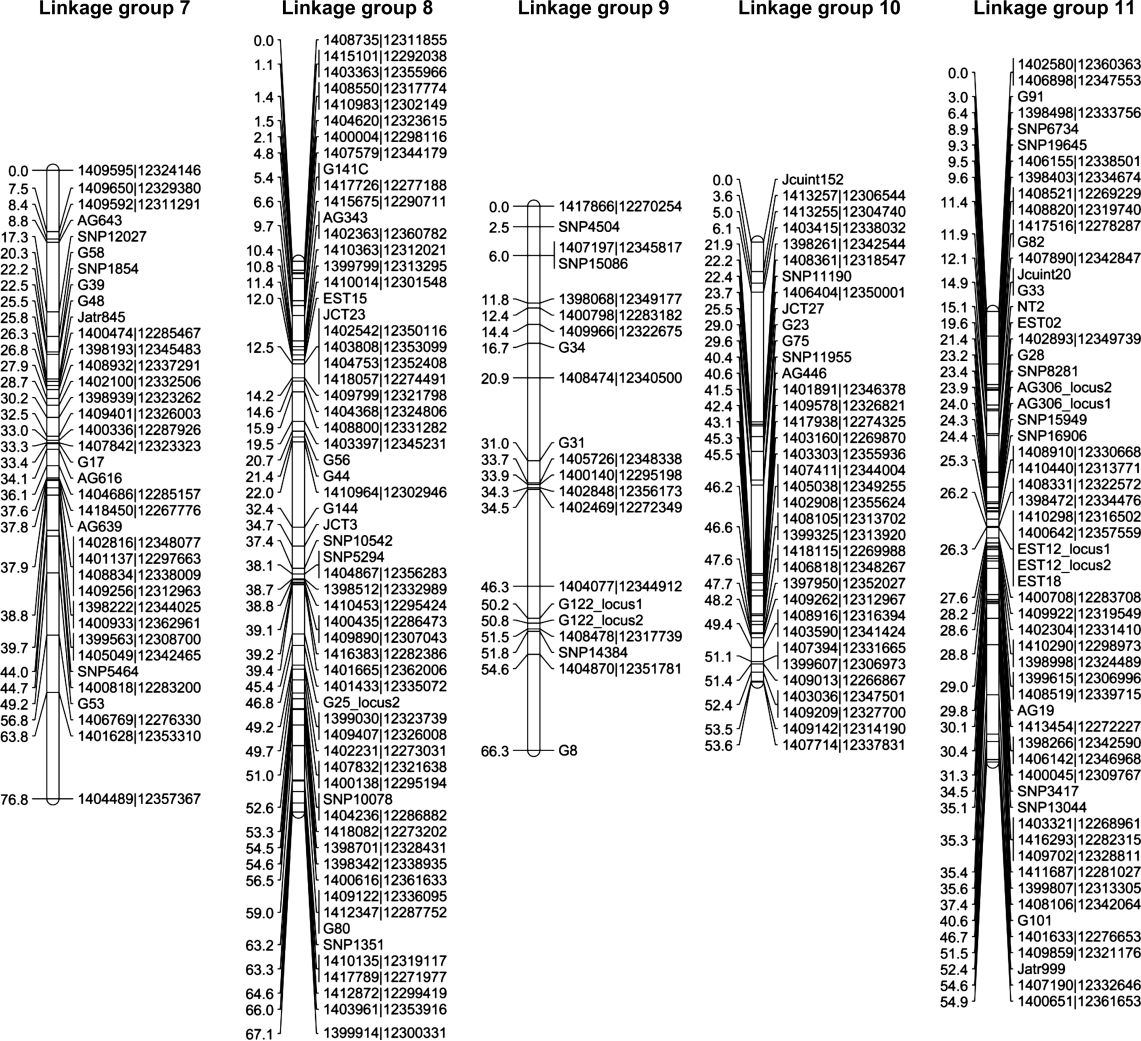
Integrated genetic map for linkage groups 7–11 of *Jatropha curcas* produced from four mapping populations. Linkage group positions are indicated in cM (Kosambi).

To build a linkage map of *J. curcas*, we used four mapping populations created from parental lines displaying differences in a range of traits as shown in Table1. Genotyping assays were performed using both SNP and SSR markers according to the Experimental procedures section. The linkage maps for each mapping population were built individually using CRI-MAP 2.503 (www.animalgenome.org), which uses the multipoint likelihood to calculate genetic distances (Lander *et al*., 1987). Each map contained 11 linkage groups, which is consistent with cytological evidence showing *J. curcas* is diploid with 22 chromosomes (*n* = 11) (Dehgan and Webster, 1979). After completion and error checking of the individual maps, the genotype files were merged and then used to build an integrated linkage map (Figures1 and 2). The total genetic distance of this integrated map was 717.0 cM, with an average marker density of 1.5 and 1.8 cM for all and unique loci, respectively. There are relatively few gaps within the integrated map, with only two pairs of loci separated by more than 15 cM, seven pairs of loci by more than 10 cM, and 30 pairs of loci by more than 5 cM. A summary of the map size, number of markers, unique loci and average marker density for each of the individual maps is shown in Table2. The individual maps are presented in File S5. Although marker densities and numbers were lower for the individual maps, the average density and length were in each case still high compared to first generation maps for most species. The least populated map (G51 × CV) still contained 253 markers and had a mean density of 3.3 cM based on unique loci. The marker coverage on all four maps is sufficient for interval mapping, where an interval of 10 cM is regarded as adequate (Mayer, 2005).

**Table 1:** F_2_ mapping populations used in this study

Mapping population parents and origin			
Maternal parent ♀	Pollen parent ♂	size	Primary traits
G33 (Guatemala)	G43 (Guatemala)	320	Seed toxicity G33 = toxic, G43 = nontoxic
G51 (Guatemala)	CV (Cape Verde)	214	Branching G51 = open branching, CV = closed branching Oil content G51 = 36.9% oil, CV = 26.0% oil
QV-JAT03 (Cape Verde)	QV-JAT02 (Mexico)	220	Oil content QV-JAT03 = 34.7% oil, QV-JAT02 = 36.5% oil
QV-JAT02 (Mexico)	QV-JAT01 (India)	220	Fatty acid composition QV-JAT02 = 34.4% oleic acid, QV-JAT01 = 42.1% oleic acid

**Table 2:** Summary of linkage group size marker number and density for the integrated map and four F_2_ populations

	Linkage group											
Map	01	02	03	04	05	06	07	08	09	10	11	Total
Integrated map												
Markers	38	34	58	45	60	52	37	62	21	36	59	502
Unique loci	29	29	47	32	50	40	32	46	20	29	44	399
Distance, cM	50.9	67.3	69.2	62.7	64.4	82.1	76.8	67.1	66.3	53.6	54.9	717.0
Mean density (all), cM	1.4	2.0	1.2	1.4	1.1	1.6	2.1	1.1	3.3	1.5	1.0	1.5
Mean density (unique), cM	1.8	2.4	1.5	2.0	1.3	2.1	2.5	1.5	3.5	1.8	1.2	1.8
QV-JAT03 × QV-JAT02												
Markers	27	15	48	33	41	35	32	51	15	34	51	382
Unique loci	21	13	33	24	32	29	26	38	13	23	30	283
Distance, cM	44.2	53.9	60.1	61.2	54.1	65.2	76.6	63.9	67.5	51.2	52.4	650.3
Mean density (all), cM	1.7	3.9	1.3	1.9	1.4	1.9	2.5	1.3	4.8	1.6	1.0	1.8
Mean density (unique), cM	2.2	4.5	1.9	2.7	1.7	2.3	3.1	1.7	5.6	2.3	1.8	2.4
QV-JAT02 × QV-JAT01												
Markers	28	14	50	30	42	37	32	50	14	34	49	380
Unique loci	22	13	38	20	30	29	27	37	13	24	30	283
Distance, cM	52.9	54.2	56.2	56.9	53.0	65.1	74.5	64.3	50.6	46.1	57.3	631.1
Mean density (all), cM	2.0	4.2	1.1	2.0	1.3	1.8	2.4	1.3	3.9	1.4	1.2	1.7
Mean density (unique), cM	2.5	4.5	1.5	3.0	1.8	2.3	2.9	1.8	42	2.0	2.0	2.3
G33 × G43												
Markers	26	23	11	26	30	35	19	27	18	18	28	261
Unique loci	18	20	9	20	25	26	15	17	17	16	23	206
Distance, cM	48.7	72.5	36.9	67.0	59.4	77.2	52.4	65.4	52.2	60.5	52.2	644.2
Mean density (all), cM	1.9	3.3	3.7	2.7	2.0	2.3	2.9	2.5	3.1	3.6	1.9	2.6
Mean density (unique), cM	2.9	3.8	4.6	3.5	2.5	3.1	3.7	4.1	3.3	4.0	2.4	3.3
G51 × CV												
Markers	23	7	31	35	35	23	18	34	5	21	21	253
Unique loci	17	6	25	22	25	16	12	28	5	10	15	181
Distance, cM	48.9	5.4	58.5	57.5	50.9	57.1	74.6	67.9	59.6	34.9	45.9	561.2
Mean density (all), cM	2.2	0.9	2.0	1.7	1.5	2.6	4.4	2.1	14.9	1.7	2.3	2.3
Mean density (unique), cM	3.1	1.1	2.4	2.7	2.1	3.8	6.8	2.5	14.9	3.9	3.3	3.3

To physically map each of the molecular markers used in this study, a blastn search was conducted against build 4.5 of the *J. curcas* draft genome sequence (www.kazusa.or.jp/jatropha) (Hirakawa *et al*., 2012). All except one of the markers used produced at least one hit against this database (File S6). Most of the markers aligned only with a single scaffold, and in some cases, multiple markers were located on the same scaffold. Using this approach, it was possible to determine the location of 3077 of the 39 277 predicted gene elements predicted within this genome assembly, and 17 Mbp of the 297 Mbp of sequence (Hirakawa *et al*., 2012). Comparative mapping of each of the *J. curcas* scaffolds against the *R. communis* draft genome was also conducted using the *promer* command of the MUMmer software (Chan *et al*., 2010; Kurtz *et al*., 2004). For the current assembly of the *J. curcas* genome, the mean and N50 scaffold lengths are 7.6 and 16.0 kbp, respectively (Hirakawa *et al*., 2012). The *R. communis* genome sequence currently has mean and N50 scaffold lengths of 14.0 and 496.5 kbp, respectively (Chan *et al*., 2010), with the mean increasing to 93.0 kbp when scaffolds less than 2 kbp are excluded. The *R. communis* genome therefore currently comprises fewer and larger scaffolds than the *J. curcas* genome. Our analysis indicated a number of large syntenic blocks on each of the chromosomes (File S6). This synteny mapping approach proved useful for fine mapping of locus controlling seed toxicity (see below). Further improvement in the scaffold lengths of the either the *J. curcas* or *R. communis* genome, or an increase in marker density of the *J. curcas* linage map would allow a more in-depth analysis of synteny between these genomes.

Previously, the only linkage map available for *J. curcas* was a map produced for an interspecific cross between *J. curcas* and *J. integerrima* (Wang *et al*., 2011). Although interspecific maps are useful tools for the location of specific genes, or the identification of beneficial traits which may be introgressed from outside the species, their potential for identifying useful traits within *J. curcas* germplasm is limited, as no assessment of the potential of alleles within the species can be made using these maps. This first intraspecific *J. curcas* map therefore represents a significant advance for this crop and will be a useful tool for marker-assisted breeding. The maps for the individual crosses have good coverage, although some arms are not mapped in two of the populations (File S5). In some instances, it will be possible to close these gaps by developing additional markers at specific locations on the map by searching for additional polymorphisms in flanking regions of the *J. curcas* genome. We have demonstrated this approach on linkage group 8 for mapping population G33 × G43 (see below). In some cases, the gaps on maps are likely to be due to identity by descent. For example, in an attempt to map the upper arm of linkage group 3 in the G33 × G43 mapping population (File S5), we screened an additional 48 markers using this approach. None were polymorphic in this population (data not shown).

The total map length of 717.0 cM is much smaller than the previously published interspecific map distance of 1440.9 cM. Based on the high number of markers we have placed on the consensus map, it is unlikely that the difference in map length is caused by a lack of markers at the ends of the linkage groups. The map distances, distances between loci and order of loci were also very consistent between each of the four mapping populations used in this study. It is therefore likely that the map distance observed in this study can be mainly attributed to differences in the error rates of the genotyping data in the two studies. Uncorrected genotyping errors can drastically increase the calculated map lengths (Hackett and Broadfoot, 2003).

Cytological studies of the *J. curcas* genome have revealed that the estimated genome size for this species is 416 Mbp (Carvalho *et al*., 2008), which is consistent with the size of the assembled genome data, which is currently 297 Mbp (Hirakawa *et al*., 2012). Based on these studies, 1 cM of this *J. curcas* map should correspond to a mean physical distance of around 0.5 Mbp.

This is the first intraspecific genetic linkage map that has been published for *J. curcas*, a plant which, although showing much promise, requires significant improvement before sustainable economic cultivation can become a reality. For a first generation map, the density is more than sufficient for trait analysis by interval mapping. This map therefore provides a valuable resource for the development of better varieties for the economic production of renewable oil.

### Phorbol ester biosynthesis in the seeds of *J. curcas* is a maternally controlled trait encoded by the nuclear genome

As a prerequisite to mapping the locus (or loci) responsible for seed toxicity, the inheritance of the PE biosynthesis trait was studied. Our previous investigation into the distribution of PE within the seeds of *J. curcas* revealed that although most of the PE was present within the endosperm of mature seeds, the highest concentration of these diterpenoids was present within the maternally derived inner layer of the seed coat referred to as the tegmen. The distribution of phorbol esters within the endosperm is also not uniform; much higher concentrations were observed in the outer (seed coat facing) endosperm layers than the inner (embryo facing) layers (He *et al*., 2011). Sujatha *et al*., 2005 have demonstrated previously that nontoxic plants pollinated by toxic plants bear nontoxic seed, and *vice versa*. Together, these observations suggest that the PE biosynthesis may only occur within the tegmen. This maternal tissue is crushed during the final stages of seed development, and the PE may then diffuse into the endosperm due to their hydrophobicity.

The phorbol ester content of seed produced from reciprocal crosses between toxic and nontoxic varieties of *J. curcas* was analysed (Table3). Genotyping of the endosperm from F_1_ to F_2_ seeds was also conducted using a subset of SSR markers to confirm that crosses were genuine (data not shown). PE^−ve^ maternal plants cross-pollinated with pollen from PE^+ve^ plants produced only seeds lacking PE, whereas PE^+ve^ maternal plants cross-pollinated with the pollen from PE^−ve^ produced seeds contain PE. Self-pollination of F_1_ plants derived from both PE^−ve^♀ × PE^+ve^ ♂ and PE^+ve^♀ × PE^−ve^ ♂ crosses resulted in F_2_ seed all of which contained PE. Open pollinated seeds were collected from F_2_ plants. Thirty one of 120 plants analysed produced seeds lacking phorbol esters (File S7). The trait did not segregate within seeds collected from an individual plant. These observations are consistent with a genetic locus causing seeds to lack phorbol esters being encoded on the nuclear genome, and the phorbol esters being synthesized only within maternal tissues. The 3 : 1 segregation also confirms that PE biosynthesis is a dominant monogenic trait.

**Table 3:** Maternal control of phorbol ester biosynthesis

Maternal parent ♀	Pollen parent ♂	Seeds	Phorbol ester content of seed
PE^+ve^	PE^+ve^	Self	Present
PE^−ve^	PE^−ve^	Self	Absent
PE^+ve^	PE^−ve^	F_1_	Present (*n* = 6)
PE^−ve^	PE^+ve^	F_1_	Absent (*n* = 12)
PE^+ve^ ♀ x PE^−ve^ ♂ F_1_	PE^+ve^ ♀ x PE^−ve^ ♂ F_1_	F_2_	Present (*n* = 8)
PE^−ve^ ♀ x PE^+ve^ ♂ F_1_	PE^−ve^ ♀ x PE^+ve^ ♂ F_1_	F_2_	Present (*n* = 8)
PE^+ve^ ♀ PE^−ve^ ♂ F_2_	Open pollinated		Individual plants exclusively bear seeds that either do or do not contain PE. PE were absent in seeds from 31/120 plants

The maternal control of phorbol ester biosynthesis has some important implications for cultivation of this crop. These data on the inheritance of phorbol ester biosynthesis indicate that as the embryo/endosperm genome does not control PE biosynthesis, there is no risk of maternal nontoxic plants bearing seed containing phorbol esters due to cross-pollination by a toxic plant. This observation means that guaranteeing production of nontoxic seeds should be feasible even in locations where genotypes producing toxic seed are growing.

### Identification of a locus controlling phorbol ester biosynthesis on linkage group 8

**Figure 3:**
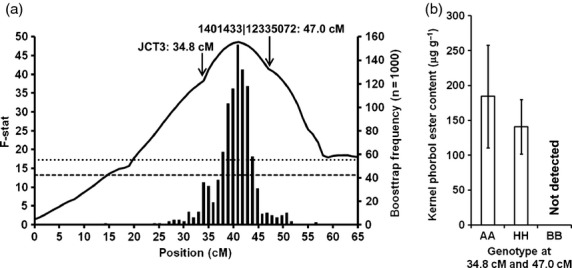
(a) QTL analysis of phorbol ester content based on seeds collected from 120 F_2_ plants in mapping population G33 × G43 for linkage group 8. *Left y axis*: The solid black line shows the *F*-stat score obtained using GridQTL. The horizontal lines show the genome-wide significance thresholds at *P *=* *0.05 (lower, dashed) and *P *=* *0.01 (upper, dotted) based on 1000 iterations. *Right axis*: The vertical bars show the QTL distribution calculated using bootstrap analysis (*n* = 1000). (b) Kernel phorbol ester content of seeds collected from F_2_ plants which were homozygous at two markers flanking the seed toxicity locus (‘AA’, G33 allele), heterozygous (‘HH’), or homozygous (‘BB’, G43). The error bars represent the standard deviations.

To conduct linkage analysis for loci controlling phorbol ester biosynthesis, the data from the 120 F_2_ plants in mapping population G33 × G43 were analysed. Although the presence of phorbol esters is a qualitative trait, the data were scored quantitatively as the actual concentration of PE present may be influenced by whether the plant is heterozygous at the PE locus. The QTL analysis was performed using GridQTL and resulted in the identification of a single locus at 41 cM on linkage group 8 of the G33 × G43 linkage map (Figure3a). The 95% confidence intervals for the QTL position were 31–49 cM (18 cM length). On the map for this population, the two flanking markers were positioned at 34.8 cM (JCT3) and 47 cM (1401433|12335072), respectively, which is a gap of 12.2 cM (File S5). Further analysis of the data confirmed that all F_2_ individuals which were homozygous for the G43 (nontoxic) alleles at both these positions yielded seeds which did not contain PE (Figure3b). This confirms that the mutation responsible for the lack of PE within nontoxic seeds must reside between these two markers. The mean values for the PE content of the F_2_ plants which were heterozygous at these two alleles were also significantly less than the plants which were homozygous for the G33 (toxic) alleles (*P *<* *0.01).

The identification of a locus controlling phorbol ester biosynthesis also permits the ‘nontoxic’ seed trait, which occurs naturally within only a limited set of germplasm, to be introgressed into other genetic backgrounds. The ability to improve the economics of *J. curcas* cultivation by allowing the meal to be converted into animal feed is also a significant advance in *J. curcas* breeding.

### Finer mapping of the locus affecting phorbol ester biosynthesis by marker transfer and comparative mapping

**Figure 4:**
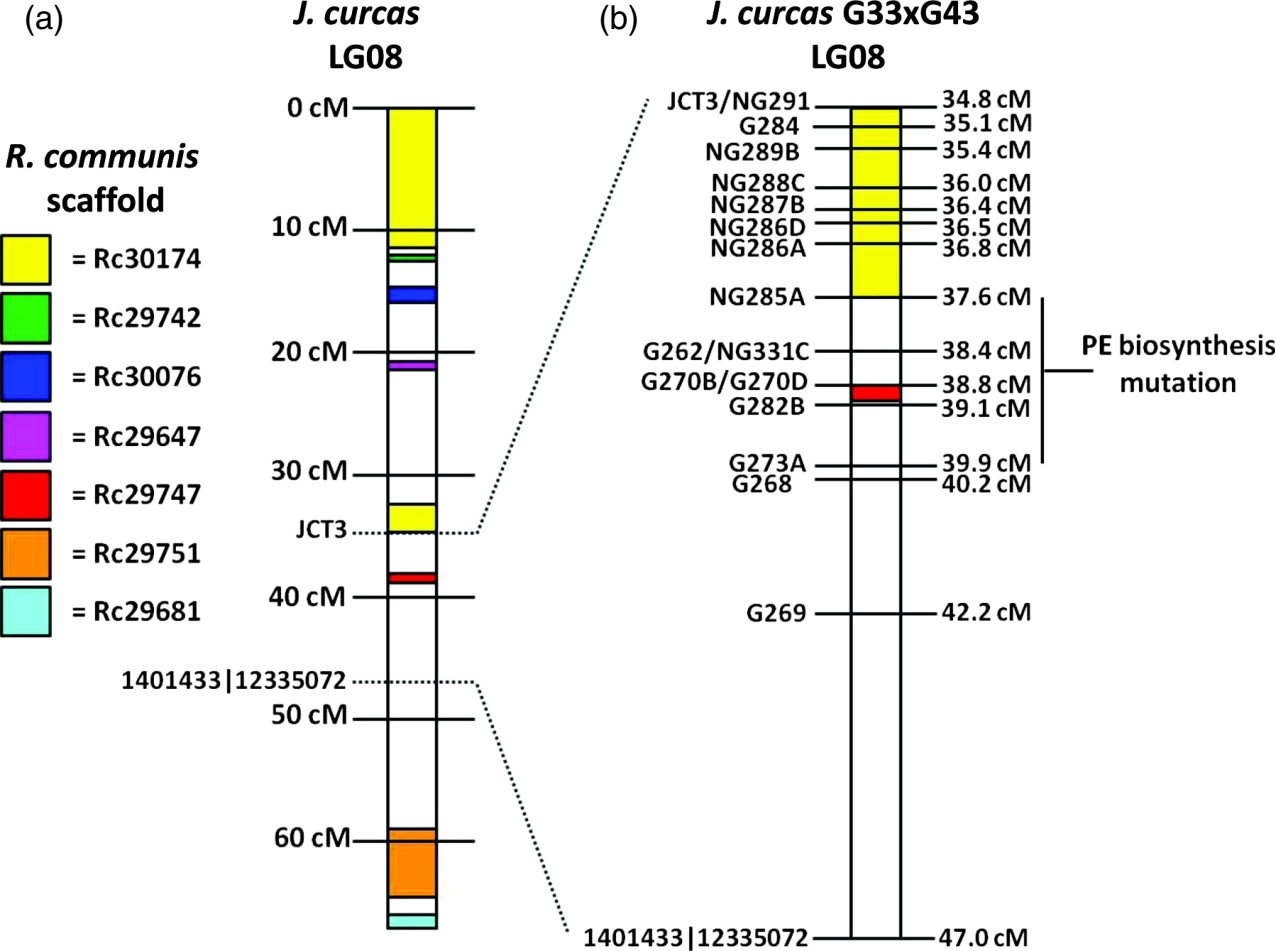
(a) Identification of syntenic regions on *J. curcas* linkage group 8 by comparison of scaffolds of the* J. curcus* and *R. communis* genome sequence. (b) – finer mapping of the region of linkage group 8 of mapping population G33 × G43 containing the QTL for phorbol ester biosynthesis.

Although variations in PE content for seeds from plants homozygous and heterozygous for the PE^+ve^ alleles were observed, seed toxicity can still be considered a dominant and qualitative trait as only plants containing two copies of the PE^+ve^ allele produced seed containing no phorbol esters. This permits the fine mapping of this trait without the problems encountered with large confidence intervals for the quantitative polygenic traits (Mangin and Goffinet, 1997). To conduct finer mapping of the locus controlling phorbol ester biosynthesis, two strategies were used. First, blastn searches were performed against the *J. curcas* genome sequence (Hirakawa *et al*., 2012; Sato *et al*., 2011) using the markers which mapped in at least one of the other populations in this study or had mapped in the interspecific cross produced previously (Wang *et al*., 2011). The scaffolds identified from these blastn searches were then mined *in-silico* for SSR sequences (Table4). Those sequences found to be polymorphic were genotyped in mapping population G33 × G43 then added to the linkage map. As a second approach to increasing the map density in the region containing the locus responsible for PE biosynthesis, a comparative mapping approach was used which exploited the microsynteny (colinearity) between the *J. curcas* and *R. communis* genomes. Where synteny exists between chromosomal regions of these two species, it is possible to infer the relative positions of *J. curcas* genes based on the larger *R. communis* assemblies. The synteny analysis revealed a number of syntenic blocks on linkage group 8 of *J. curcas* (Figure4, Table4 and File S6). In particular, the *R. communis* scaffold 30 174 mapped to two regions of *J. curcas* linkage group 8, one of which was located near the locus for PE biosynthesis. A number of additional SSR markers were created from *J. curcas* scaffolds which were syntenic with this *R. communis* scaffold (Table4). The result from the mapping of 16 additional markers in the region containing the locus is shown in Figure4. Analysis of the seed PE content data with the genotyping data indicated that the genetic locus conferring the absence of PE biosynthesis resides between makers NG285A and G273A. The genetic distance of this region is 2.3 cM. Our ability to map this trait to less than this distance is limited by a breakdown in synteny to the castor genome in this region (Table4 and File S6) and a lack of further progeny with informative recombination events in this region. Our future work on the mapping of this trait will therefore include the analysis of additional F_2_ and F_3_ plants to detect additional informative recombination events in this region. A contiguous sequence for this region will also be obtained by BAC sequencing.

**Table 4:** Development of additional markers for fine mapping

Position (cM)	*Jatropha curcas* marker	Bridge marker used	*J. curcas* genome sequence		*R. communis* genome sequence	
			Scaffold	Marker position	Scaffold	Syntenic region
34.8	NG291	N/A	Jcr4S04633	10 436–10 735	30 174	2 724 054–2 749 754
34.8	JCT3	N/A	Jcr4S01892	18 299–18 382	30 174	2 695 280–2 722 645
35.1	G284	N/A	Jcr4S00160	23 304–23 696	30 174	2 663 765–2 686 270
35.4	NG289B	N/A	Jcr4S03474	13 586–13 985	30 174	2 642 521–2 666 724
36.0	NG288C	N/A	Jcr4S00541	36 116–36 276	30 174	2 553 645–2 632 854
36.4	NG287B	N/A	Jcr4S04231	31 626–31 926	30 174	2 521 238–2 536 532
36.5	NG286D	N/A	Jcr4S00188	39 908–40 226	30 174	2 442 869–2 507 050
36.8	NG286A	N/A	Jcr4S00188	10 227–10 414	″	″
37.6	NG285A	N/A	Jcr4S00012	9750–10 085	30 174	2 278 471–2 410 014
38.4	G262	SNP10542	Jcr4S05837	4261–4517	29 996	51 566–62 602
38.4	NG331C	N/A	Jcr4S01963	25 834–26 225	29 996	62 985–63 609
38.8	G270B	1404867|12356283	Jcr4S03364	6780–6962	29 747	274 981–367 675
38.8	G270D	1404867|12356283	Jcr4S03364	17 347–17 502	″	″
39.1	G282B	SNP5294	Jcr4S01263	23 937–24 259	Low level of synteny	
39.9	G273A	1400435|12286473	Jcr4S08292	1249–1548	Low level of synteny	
40.2	G268	1401665|12362006	Jcr4S01025	42 898–43 155	28 266	19 280–78 337
42.2	G269	eSNP0195	Jcr4S01910	8711–8987	Low level of synteny	
47.0	1401433|12335072	N/A	Jcr4S02123	25 922–25 718	Low level of synteny	

The mapping of the mutation responsible for the lack of PE biosynthesis to within a relatively small region of the genome means that it will now be possible to breed new high oil yielding varieties of *J. curcas* which lack phorbol esters. This 2.3 cM region is likely to represent less than 1% of the entire 717.0 cM genome, which means it will be possible to remove large portions of the remaining genome of the nontoxic variety, which may contain many undesirable alleles, by backcrossing.

We have also demonstrated the utility of exploiting the existence of syntenous colinear regions within genomes to develop markers targeted to specific regions of a genome. Although this comparative mapping has been used previously in organisms such as *Brassica* sp. (Lagercrantz *et al*., 1996) and *Triticum aestivum* (Liu and Anderson, 2003), this approach has typically relied on the availability of a near-complete genome sequence (i.e. *Arabidopsis thaliana* and *Oryza sativa*, respectively). In this study, we compared genomic sequences of *J. curcas* (Hirakawa *et al*., 2012; Sato *et al*., 2011) and *R. communis* (Chan *et al*., 2010), both of which are in a draft stage, containing thousands of scaffolds. Due to the low cost and rapidity of the shotgun sequencing approach, most genome sequences are currently only assembled as ‘drafts’. This approach to mapping will therefore have utility in other species.

The four populations in this study were also developed from parents with variations in a number of traits including oil content and seed size. We will therefore use these mapping populations to dissect these traits once these perennial plants approach maturity.

## Experimental procedures

### Mapping populations

Four mapping populations were used in this study, as detailed in Table1. Parental lines G43, Cabo Verde (CV), QV-JAT01, QV-JAT02 and QV-JAT03 were homozygous at all loci, whereas parental lines G33 and G51 were heterozygous at some of the loci scored in this study. To minimize the number of uninformative markers in mapping populations G33 × G43 and G51 × CV, the F_2_ were created using an F_1_ intercross. All other mapping populations were created by self-pollinating F_1_ plants.

### DNA extraction and amplification

DNA from mapping populations G33 × G43 and G51 × CV was extracted using the Qiagen DNEasy Plant Mini kit (Qiagen, Venlo, the Netherlands). DNA from the other mapping populations was extracted using the CTAB protocol (Doyle and Doyle, 1987). DNA was quantified using the DNA binding dye EvaGreen (Biotium, Hayward, CA), using salmon sperm DNA as a standard. (Wang *et al*., 2006). Where insufficient DNA was obtained, DNA was amplified using the Qiagen RepliG kit.

### Single Nucleotide Polymorphism (SNP) marker identification and analysis

SNPs were obtained from two sources. A reduced representation library was produced from toxic and nontoxic provenances of *J. curcas* using the CRoPS® technique (van Orsouw *et al*., 2007). SNPs identified by this technique were converted to an Illumina VeraCode assay (File S1). SNPs were also identified from ESTs of developing seeds obtained from pyrosequencing of cDNA from varieties bearing toxic (King *et al*., 2011) and nontoxic seeds (A.J. King and I.A. Graham, unpublished). The SNPs obtained from the EST databases were assayed using one of two different methods. *Capillary electrophoresis based method;* an additional three noncomplementary nucleotides were added to the 5′-end of one of the allele-specific primers to permit allele discrimination by amplicon size. An additional mismatch near the 3′-end of each allele-specific primer was also incorporated to increase specificity (Bui and Liu, 2009). An M13 tail sequence (5′-TGTAAAACGACGGCCAGT-3′) was added to the 5′-end of the locus-specific primers to facilitate fluorescent labelling of the PCR products. PCR reactions for the two alleles were performed independently using the Qiagen Multiplex PCR kit. Each 10 μL reaction contained 5 μL of reagent, 2–20 ng of DNA, 0.5 pmol allele-specific primer, 0.5 pmol locus-specific primer and 2 pmol of either FAM (‘low’ allele) or VIC (‘high’ allele) labelled M13 primer. Up to 12 SNPs were multiplexed per PCR reaction. After pooling of the ‘low’ and ‘high’ allele PCR reactions, fragment analysis was performed using an Applied Biosystems 3730 DNA Analyzer (Life Technolgies, Carlsbad, CA) using a LIZ-500 internal standard. *KASPar method*; the KASPar assay was performed according to the manufacturer's protocol (KBiosciences, Hoddesdon, UK). Allele discrimination was performed using an Applied Biosystems 7300 Real-Time PCR System. Sequences of SNPs and the primers used for their detection by capillary electrophoresis or KASPar assays are detailed in File S2.

### Simple Sequence Repeat (SSR) marker identification and analysis

SSR markers were obtained from a number of sources as detailed in File S3. Accession numbers KC344792–KC344818 were obtained by dye-terminator sequencing clones from an AG_n_-enriched DNA library created according to the FIASCO protocol (Zane *et al*., 2002). SSRs were also identified by mining of EST data (King *et al*., 2011) or genomic sequence data (Lee and Sonnhammer, 2003). Finally, a number of publicly available SSR markers were also used. Primers for SSR analysis were designed using Primer3Plus (Untergasser *et al*., 2007) according to the nearest-neighbour method (SantaLucia, 1998). An M13 tail sequence was appended to 5′-end of the shortest primer to facilitate fluorescent labelling of the PCR products. SSR analysis was conducted using the QIAGEN Type-It Microsatellite PCR kit. Each 10 μL reaction contained 5 μL of reagent, 2–20 ng of DNA, 0.5 pmol forward primer, 0.5 pmol reverse primer and 2 pmol of VIC labelled M13 primer. Thermocycling was conducted according to the manufacturer's recommended protocol, using an annealing temperature of 57 °C. Up to 12 SSRs were multiplexed per PCR reaction. Fragment analysis was performed using an Applied Biosystems 3730 DNA Analyzer (Life Technolgies). A LIZ-500 dye was used as an internal size standard.

### Construction of genetic linkage maps

To build linkage maps, CRI-MAP version 2.503 was used (Lander and Green, 1987). Linkage maps for each of the mapping populations were first constructed independently. Linkage groups were first determined using the *twopoint* function with an LOD cut-off point >4.0. Maps for each linkage group were then constructed using the *build* function. Orders were checked with the *flipsn* function. To check for genotyping errors, the *chrompic* function was used to identify suspect double crossover events—that is, those observed within a short distance (<5 cM). Suspect markers were then checked where possible by reanalysis of the original data and corrected when a manual scoring error was evident. After the initial correction, any markers still found to produce excessive double-crossovers (>2.5% of population) were omitted from the map. The remaining suspect genotypes resulting in double-crossovers were converted to missing data. After completion of the four individual linkage maps, the genotype files were combined manually using Microsoft Excel spreadsheets. An integrated linkage map was then produced by rebuilding a single map using CRI-MAP.

### Analysis of phorbol ester content of seeds

The testa of the seeds, which have previously been shown to lack phorbol esters (He *et al*., 2011), was removed and the kernels were ground to a fine powder in a mortar and pestle. Approximately 200 mg of kernel was transferred to a 2 mL Eppendorf tube. Five micro-liter of 1 mg/mL of phorbol 12-myristate a 13-acetate (Sigma-Aldrich Co Ltd, Poole, Dorset, UK) was added as an internal standard. The seed kernel was then extracted with 1.5 mL of methanol for 1 h. Solvent was recovered after centrifugation of samples at 10 000 ***g*** for 2 min. The seed kernel was then extracted twice more for 1 h with 1 mL of methanol. The extracts were combined, and the solvent was removed using a Speedvac. The oily residue was dissolved in 800 μL of hexane. Eight hundred micro-liter of acetonitrile was then added, and the sample was vortexed. After centrifugation of the sample for 1 min at 10 000 ***g***, the lower acetonitrile phase containing the phorbol esters was recovered and filtered through a 0.45 μm PTFE membrane (Millex®-LH). The filtered extract was then evaporated using a Genevac EZ-2 plus evaporator (Genevac Ltd, Ipswich, Suffolk, UK) and dissolved in 200 μL of acetonitrile. HPLC analysis was then performed as described previously (He *et al*., 2011).

### Marker-trait association studies

To determine the position of markers affecting PE biosynthesis, PE content data were scored quantitatively using GridQTL (Seaton *et al*., 2006). Chromosome-wide permutation studies were also conducted with 1000 iterations to determine significance thresholds. After the identification of the region controlling phorbol ester biosynthesis, further data analyses were performed by scoring of PE biosynthesis as a qualitative trait, by direct comparison of genotype and trait data.

### Mapping of markers onto the draft genome sequence of *J. curcas* and synteny analysis with *R. communis*

A blastn search for all markers described in this study was performed against build 4.5 of the *J. curcas* genome (Hirakawa *et al*., 2012). To identify synteny between the *J. curcas* and *R. communis* genomes, the blast2 search data against *R. communis*, which is provided on the *J. curcas* genome database, were retrieved. Where synteny was observed for a number of consecutive markers, a more detailed analysis of synteny was obtained using the *promer* function of MUMmer 3.0 (Kurtz *et al*., 2004), with the amino acid identity threshold set at 40% and the minimum length set at 100 base pairs.

## Supporting Information

**File S1** SNP markers developed from genomic DNA using the CRoPS® technique.

**File S2** SNP markers mined from seed transcriptome data.

**File S3** SSR markers used in this study.

**File S4** Markers used to bridge the maps produced in this study with the previously published map produced from an interspecific cross between J. curcas and J. integerrima (Wang.,).

**File S5** Individual linkage maps from the four mapping populations used in this study.

**File S6** Location of mapped markers on the genome sequence of J. curcas [build 4.5 (Hirakawa.,)], and syntenic blocks observed with R. communis genome.

**File S7** PE concentration measured in open-pollinated seed collected from 120 F_2_ plants.
