# Supplementary material for: Linkage mapping in the oilseed crop Jatropha curcas L. reveals a locus controlling the biosynthesis of phorbol esters which cause seed toxicity
Source: Plant Biotechnol J. 2013 Jul 30;11(8):986–96. doi: 10.1111/pbi.12092 (PMC4274016; doi:10.1111/pbi.12092)

Supplementary File 5 – Single and integrated maps

Linkage Group 1

integrated | QV-JAT03 x QV-JAT02 | QV-JAT02 x QV-JAT01 | G33 x G43 | G51 x CV

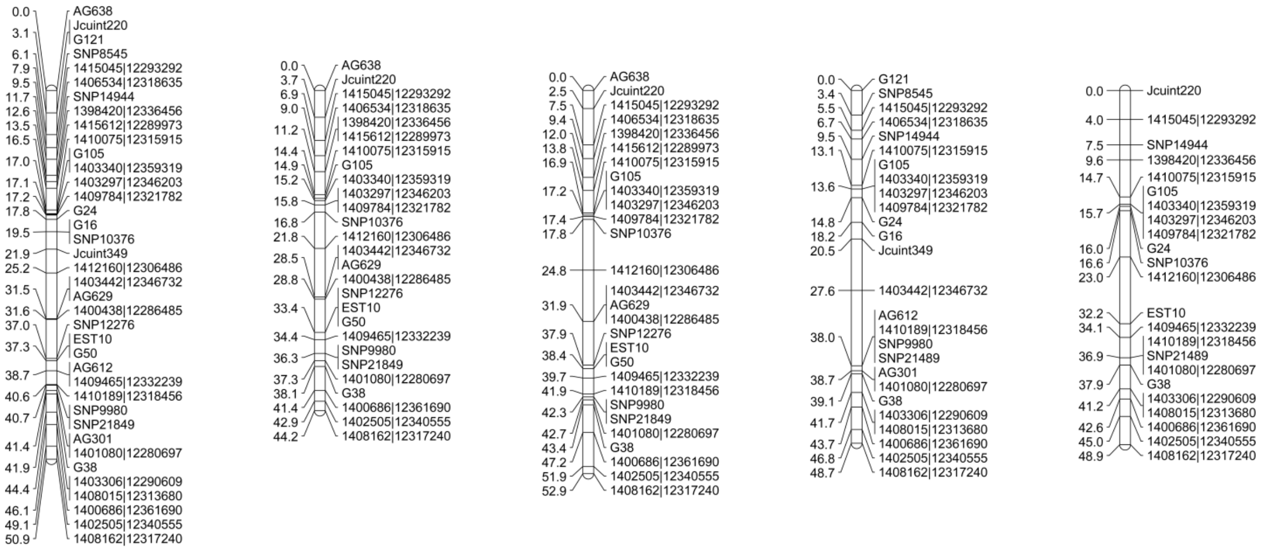

Linkage Group 2

integrated | QV-JAT03 x QV-JAT02 | QV-JAT02 x QV-JAT01 | G33 x G43 | G51 x CV

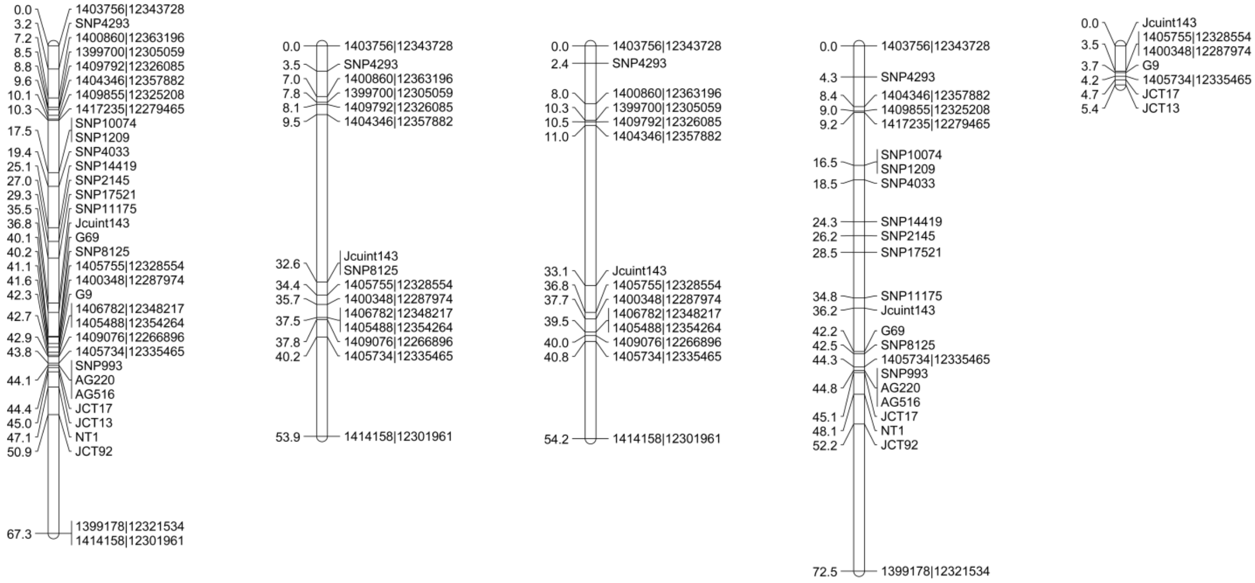

Supplementary File 5 continued – Single and integrated maps

Linkage Group 3

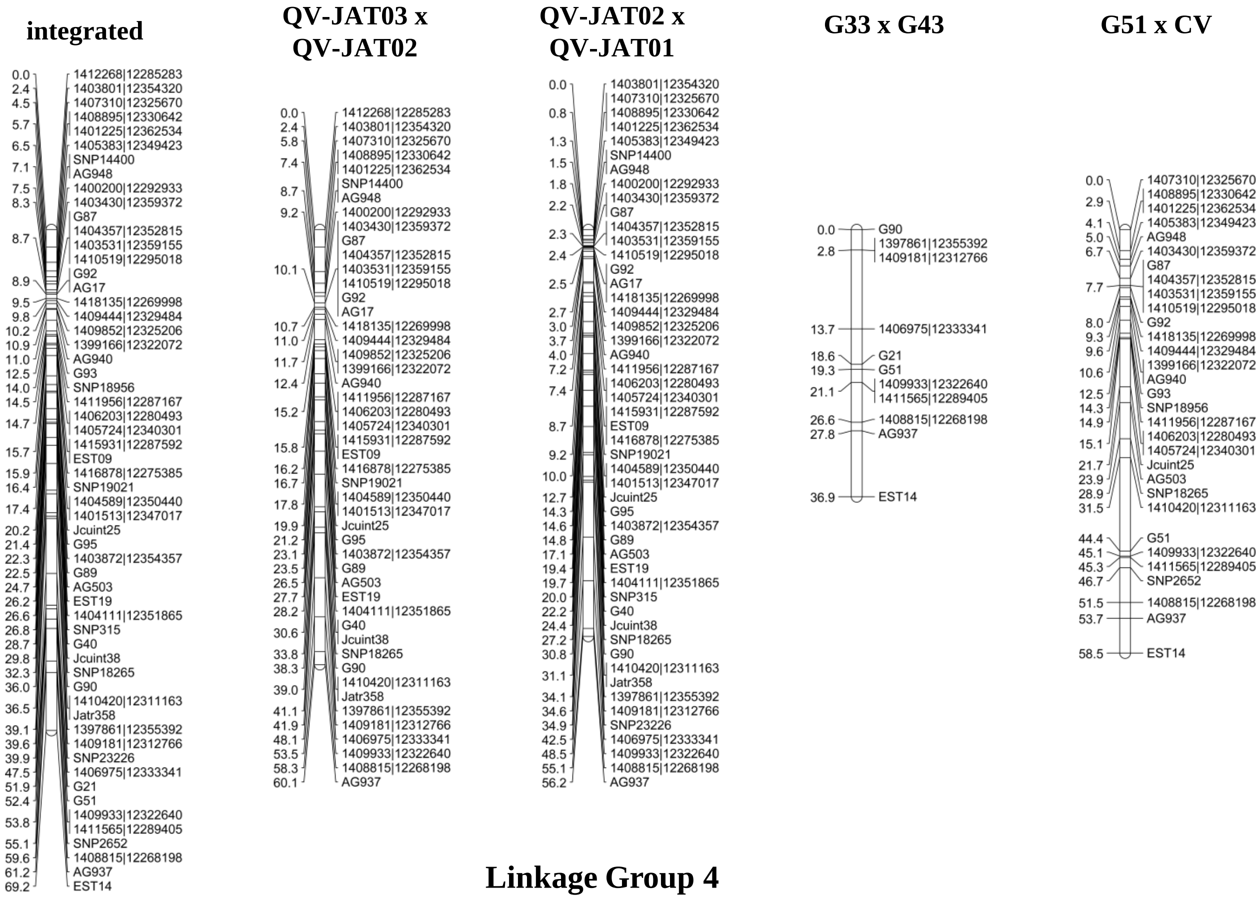

Linkage Group 4

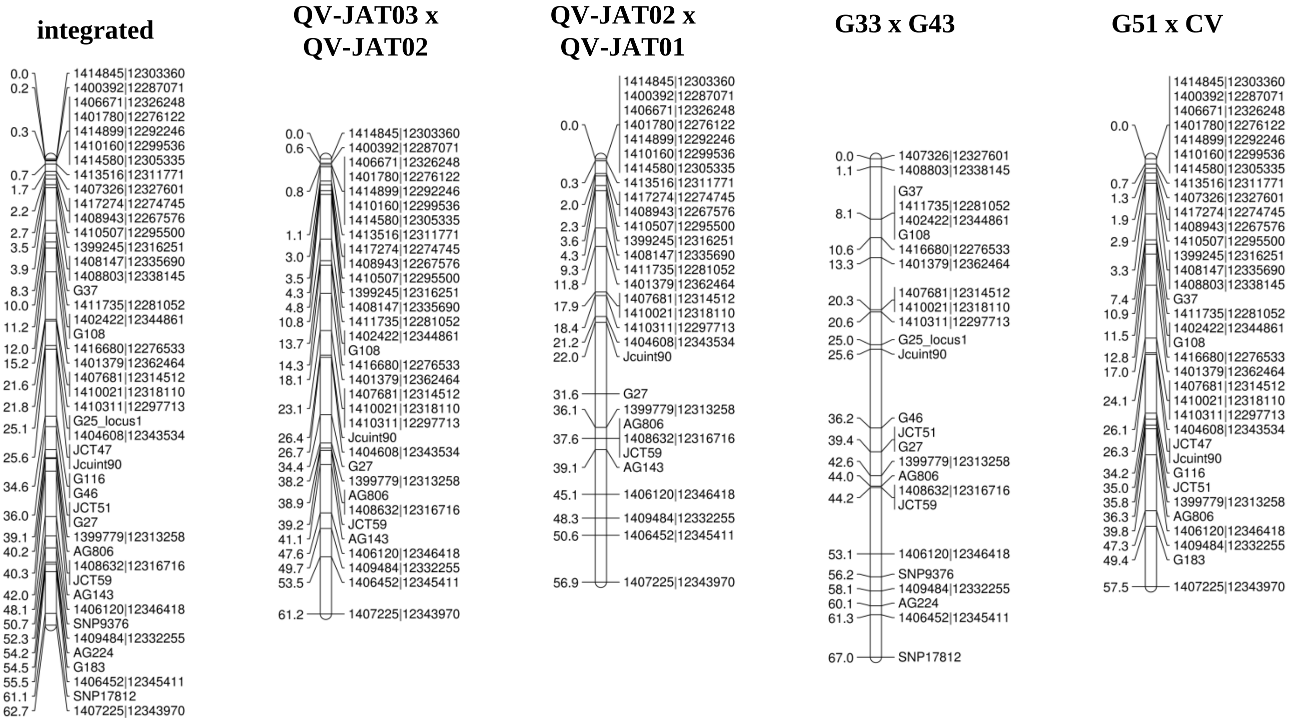

Supplementary File 5 continued – Single and integrated maps

Linkage Group 5

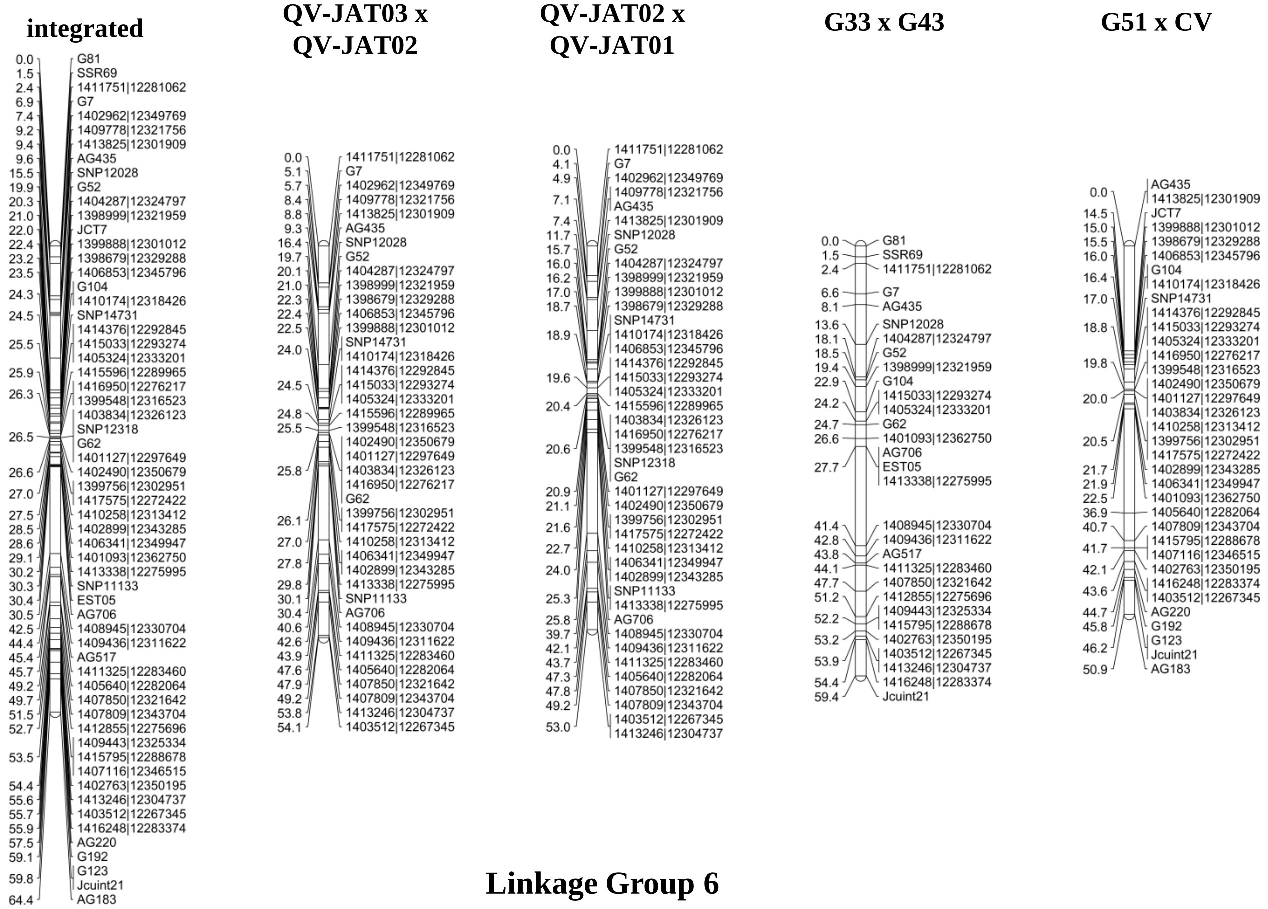

Linkage Group 6

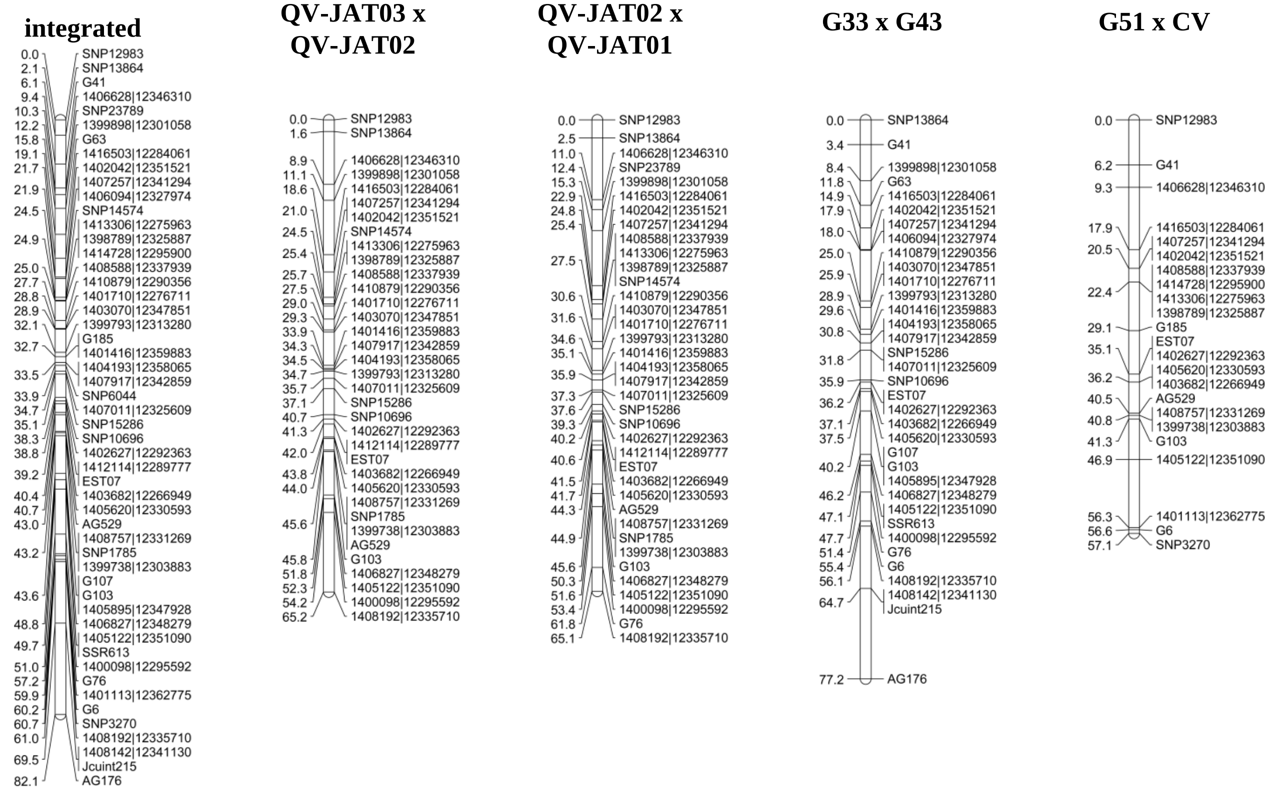

Supplementary File 5 continued – Single and integrated maps

Linkage Group 7

integrated | **QV-JAT03 x QV-JAT02** | **QV-JAT02 x QV-JAT01** | **G33 x G43** | **G51 x CV**

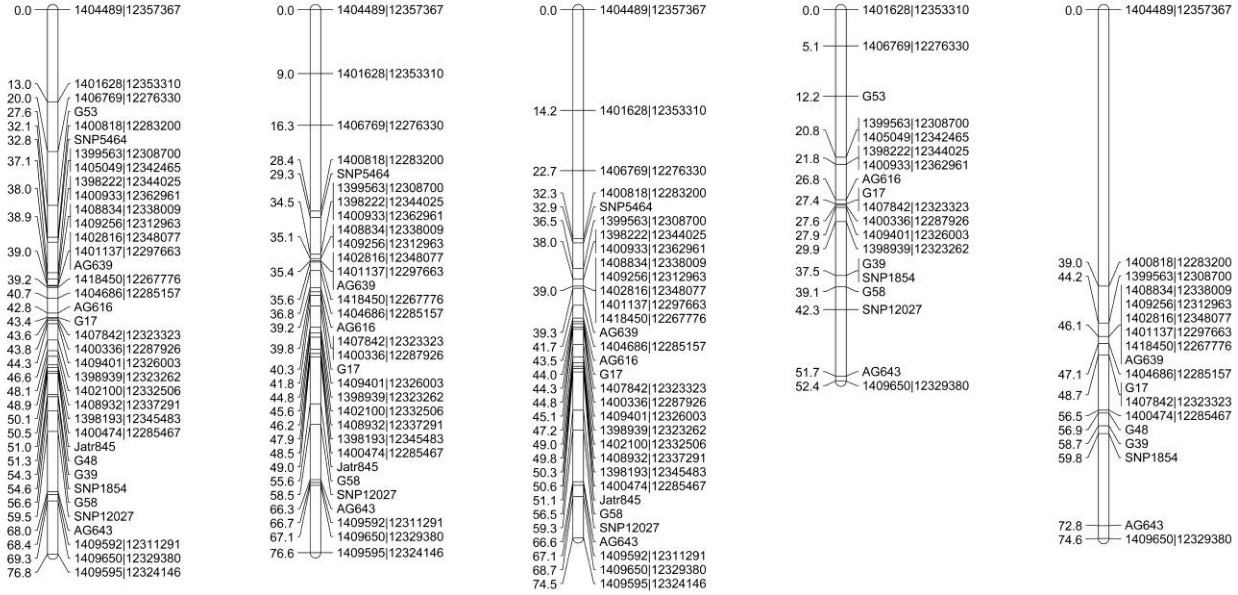

Linkage Group 8

integrated | **QV-JAT03 x QV-JAT02** | **QV-JAT02 x QV-JAT01** | **G33 x G43** | **G51 x CV**

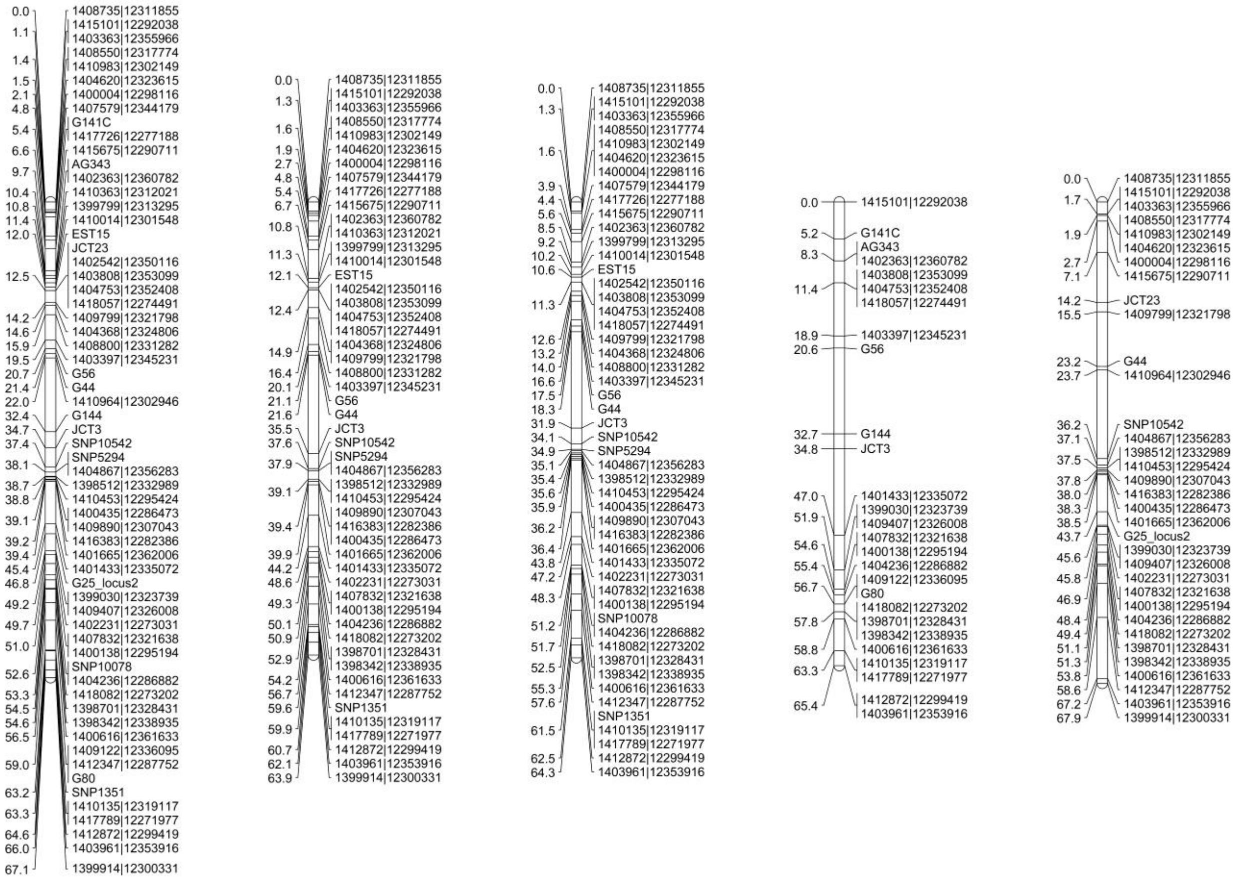

Supplementary File 5 continued – Single and integrated maps

Linkage Group 9

integrated | **QV-JAT03 x QV-JAT02** | **QV-JAT02 x QV-JAT01** | **G33 x G43** | **G51 x CV**

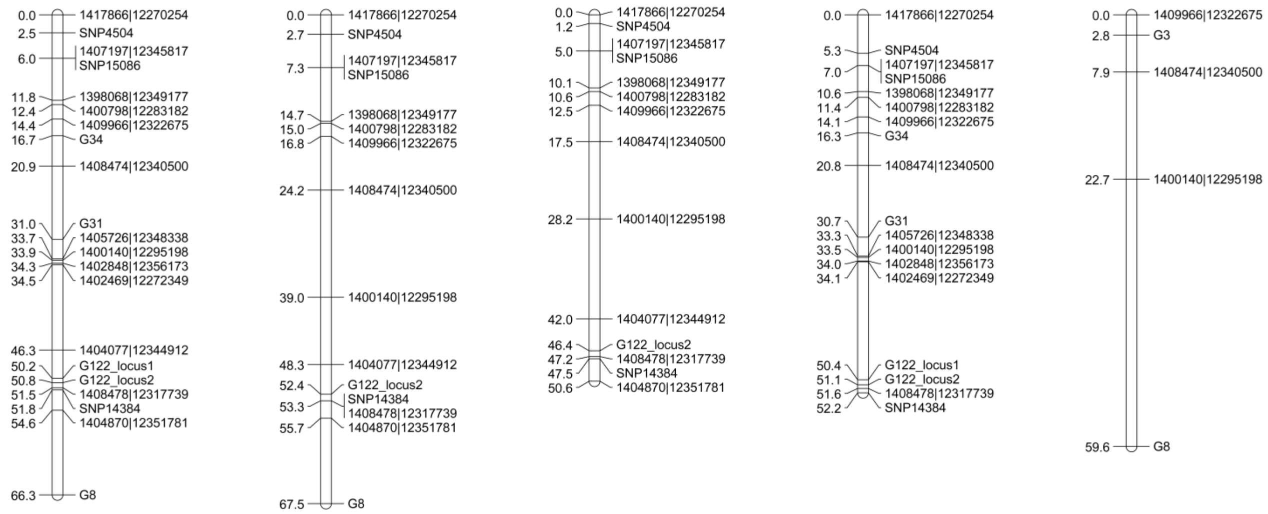

Linkage Group 10

integrated | **QV-JAT03 x QV-JAT02** | **QV-JAT02 x QV-JAT01** | **G33 x G43** | **G51 x CV**

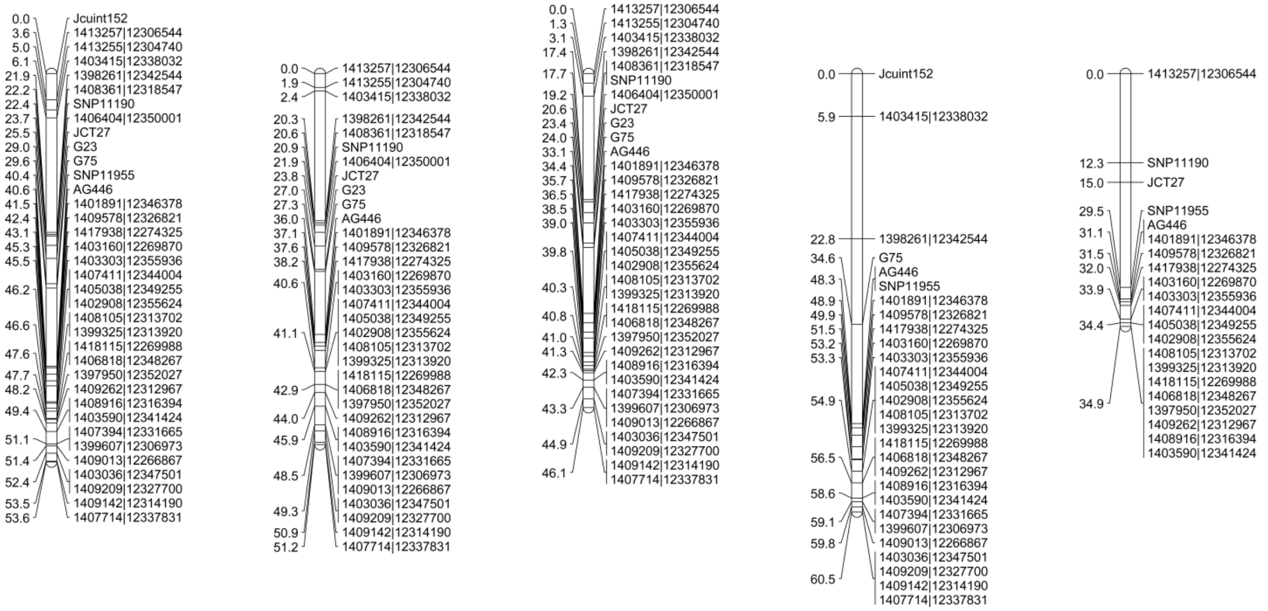

Supplementary File 5 continued – Single and integrated maps

Linkage Group 11

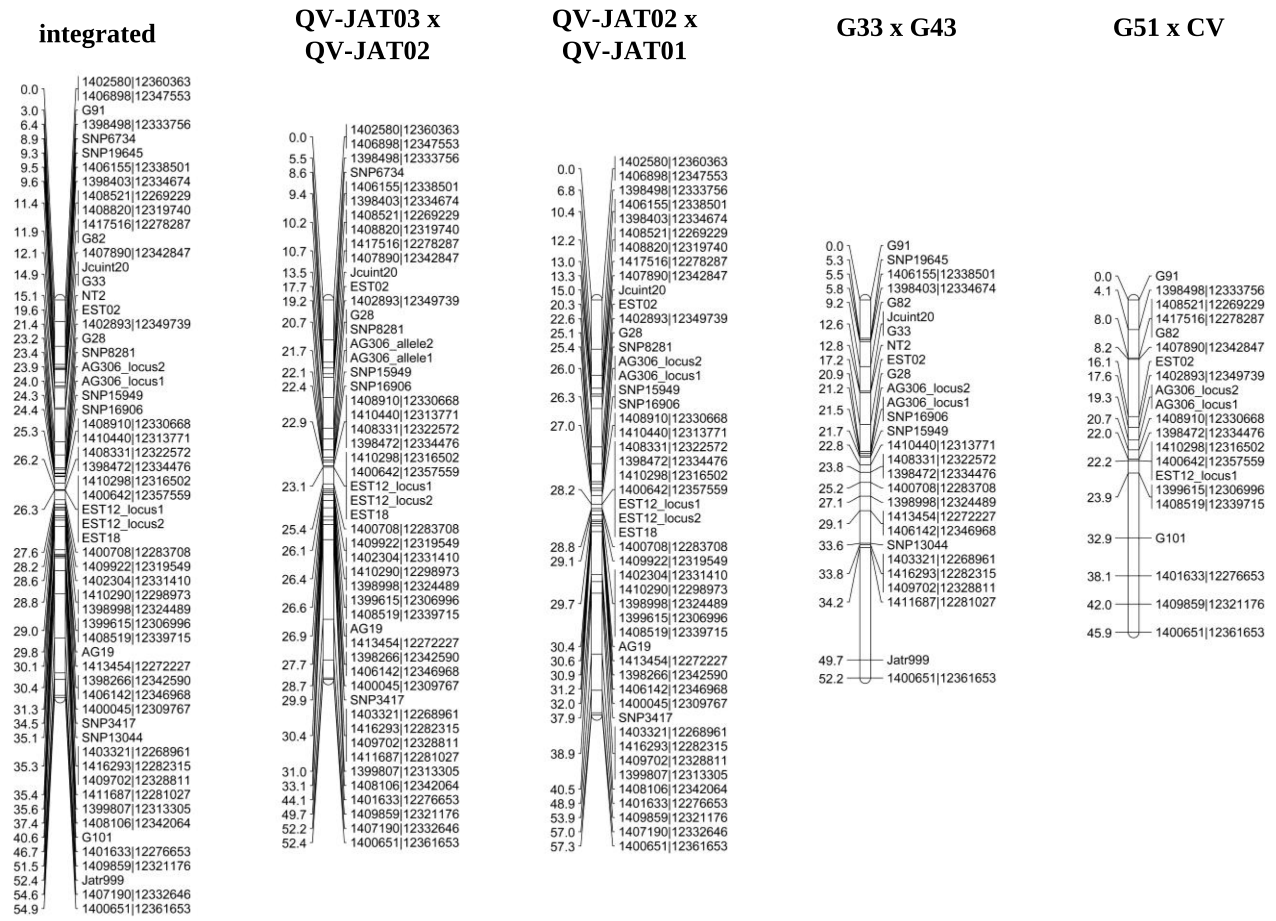

Supplement: Supplementary file 5 — File S5 Individual linkage maps from the four mapping populations used in this study. [file pbi0011-0986-sd5.pdf]
