# Supplementary material for: Linkage mapping in the oilseed crop Jatropha curcas L. reveals a locus controlling the biosynthesis of phorbol esters which cause seed toxicity
Source: Plant Biotechnol J. 2013 Jul 30;11(8):986–96. doi: 10.1111/pbi.12092 (PMC4274016; doi:10.1111/pbi.12092)
Supplement: Supplementary file 7 — File S7 PE concentration measured in open-pollinated seed collected from 120 F2 plants. [file pbi0011-0986-sd7.docx]

**Supplementary Figure 7 – PE concentration of open-pollinated seed collected from a subset of F_2_ plants of mapping population G33 x G43**

**
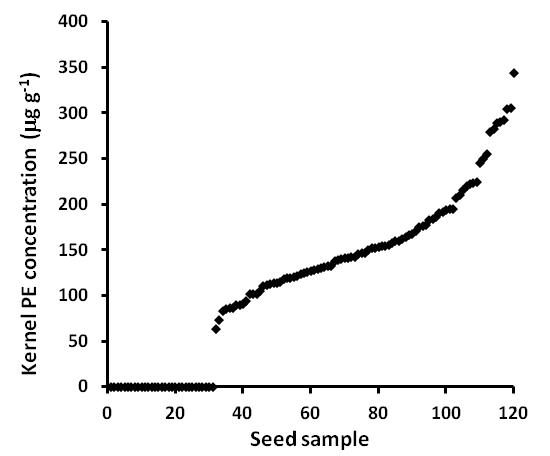
**
